# The Etiology and Treatment of Inflammation of the Uterine Appendages

**Published:** 1895-07

**Authors:** 


### Abstracts and Selections.

---

### The Etiology and Teatment of Inflammation of the Uterine Appendages.

Dr. Augustin H. Goelet, of New York, read a paper on this subject at the recent meeting of the American Medical Association at Baltimore, in which he stated that the contention was not that these inflammations of the tubes and ovaries can always be cured, but that it is frequently possible, and, unless immediate operative interference is absolutely demanded, the patient should be given the chance, and the attempt should be made before submitting her to a radical operation. This he thought particularly important, since treatment directed toward attaining this end did not militate against a subsequent operation for their removal, should it become necessary, but, on the contrary, improved the chances of an ultimate successful result. He called attention to the fact, that when once removed, these organs can not be replaced, and asked the question, if it was not a serious error, in the light of recent developments in the etiology and pathology of the inflammation of the appendages, to remove these organs without previous attempt at a cure or removal of the cause which may be operating to maintain such condition. It may be denied that diseased tubes and ovaries are removed unnecessarily, but it must be admitted that they are too often removed for disease which is amenable to patient and persistent treatment, or which may be cured by a minor surgical operation, involving no risk, such as currettage or repair of a lacerated cervix.

If these cases are submitted to careful treatment, instituted for the purpose of clearing up the surrounding exudation, and favoring drainage through the natural channel (uterus), in many instances the necessity for a radical operation would be removed, and the woman would be restored to a life of usefulness and happiness.

In corroboration of these views he reported twelve selected cases which had come to him from other gynecologists, who believed that removal of the diseased organs was the only method to be adopted for restoration of their health, yet these patients recovered completely without the loss of thse organs.

The writer stated that these were not the only cases with such an unfavorable outlook which he had been able to cure in this manner, but they had been selected from among a number of others, because they had consulted other gynecologists before they came under his observation.

